# Efavirenz use during pregnancy and for women of child-bearing potential

**DOI:** 10.1186/1742-6405-3-11

**Published:** 2006-04-07

**Authors:** Matthew F Chersich, Michael F Urban, Francois WD Venter, Tina Wessels, Amanda Krause, Glenda E Gray, Stanley Luchters, Dennis L Viljoen

**Affiliations:** 1 Epidemiologist and Statistician, International Centre for Reproductive Health, Mombasa, Kenya; 2 Fellow in Medical Genetics, Department of Human Genetics, National Health, Laboratory Service and University of Witwatersrand, Johannesburg, South Africa; 3 Clinical Director, Esselen Street Project, Reproductive Health and HIV Research Unit, University of the Witwatersrand Johannesburg, South Africa; 4 Genetic counselor, Genetic Counselling Clinic, National Health Laboratory Service & University of the Witwatersrand, Johannesburg, South Africa; 5 Professor, Department of Human Genetics, National Health Laboratory Service and University of Witwatersrand, Johannesburg, South Africa; 6 Director, Perinatal HIV Research Unit, University of Witwatersrand, Johannesburg, South Africa; 7 Field Director, International Centre for Reproductive Health, Mombasa, Kenya; 8 Professor and Head of Department of Human Genetics, National Health Laboratory Service and University of Witwatersrand, Johannesburg, South Africa

## Abstract

**Background:**

Efavirenz is the preferred non-nucleoside reverse transcriptase inhibitor for first-line antiretroviral treatment in many countries. For women of childbearing potential, advantages of efavirenz are balanced by concerns that it is teratogenic. This paper reviews evidence of efavirenz teratogenicity and considers implications in common clinical scenarios.

**Findings:**

Concerns of efavirenz-induced fetal effects stem from animal studies, although the predictive value of animal data for humans is unknown. Four retrospective cases of central nervous system birth defects in infants with first trimester exposure to efavirenz have been interpreted as being consistent with animal data. In a prospective pregnancy registry, which is subject to fewer potential biases, no increase was detected in overall risk of birth defects following exposure to efavirenz in the first-trimester.

**Discussion:**

For women planning a pregnancy or not using contraception, efavirenz should be avoided if alternatives are available. According to WHO guidelines for resource-constrained settings, benefits of efavirenz are likely to outweigh risks for women using contraception. Women who become pregnant while receiving efavirenz often consider drug substitution or temporarily suspending treatment. Both options have substantial risks for maternal and fetal health which, we argue, appear unjustified after the critical period of organogenesis (3–8 weeks post-conception). Efavirenz-based triple regimens, initiated after the first trimester of pregnancy and discontinued after childbirth, are potentially an important alternative for reducing mother-to-child transmission in pregnant women who do not yet require antiretroviral treatment.

**Conclusion:**

Current recommendations for care for women who become pregnant while receiving efavirenz may need to be re-considered, particularly in settings with limited alternative drugs and laboratory monitoring. With current data limitations, additional adequately powered prospective studies are needed.

## Background

An increasing number of women worldwide are benefiting from expanding access to antiretroviral treatment, allaying initial concerns that women would have inequitable access to treatment. In sub-Saharan Africa nearly six out of ten adults receiving antiretroviral (ARV) treatment are women, an equitable distribution as more women are infected than men [1]. A substantial proportion of these women will plan to conceive or have unintended pregnancies [2]. This raises concerns of potential ARV-induced fetal effects. Such concerns often require women and their clinicians to make trade-offs between reproductive and treatment choices.

Efavirenz (EFV) is the preferred non-nucleoside reverse transcriptase inhibitor (NNRTI) in many countries [3-5] as it is less hepatotoxic than nevirapine (NVP), does not require dose adjustment and can be used concomitantly with tuberculosis treatment. However, for women of reproductive potential these advantages are balanced by concerns that EFV increases risk for birth defects.

This paper reviews evidence of EFV teratogenicity and considers implications for common clinical scenarios.

## Evidence of efavirenz teratogenesis

Pregnant women are actively excluded from clinical trials during drug development. Assessment of drug safety in pregnancy is therefore based on less rigorous evidence, such as reproductive toxicology studies in small mammals and non-human primates, retrospective case reports and pregnancy registry data.

Concerns of EFV-induced fetal effects began after a trial with cynomolgus monkeys [6]. In the trial, monkeys were exposed to EFV throughout pregnancy at plasma drug concentrations similar to humans receiving 600 mg EFV per day. No major congenital malformations were observed in 20 control infant monkeys, but 3 of 20 EFV-exposed infants had significant abnormalities [6]. Anencephaly and unilateral anophthalmia were observed in one monkey infant, microphthalmia in another and cleft palate in a third. An increase in fetal resorptions was observed in rats given EFV, but no significant teratologic findings were reported in studies with pregnant rabbits treated with EFV [7]. Thus far in humans, four retrospective cases of central nervous system (CNS) defects in infants with first trimester exposure to EFV have been reported (three infants with meningomyelocele and one with a Dandy-Walker malformation) [7-9].

The predictive value of animal studies for humans is unknown; making it difficult for health workers to translate animal risks into an assessment of teratogenic risk in their patients. Many associations have ultimately been shown to be false positive in humans and in some instances drug testing in animals has been negative and the drug subsequently shown to be teratogenic in humans [10]. Of approximately 1 200 animal teratogens, only about 30 are known to be teratogenic in humans [11]. However, the EFV animal studies are particular concerning as abnormalities were observed in primates at drug levels comparable to therapeutic ranges in humans and positive findings were detected in more than one animal species.

The retrospective case reports in humans are difficult to interpret as neural tube defects are among the commonest birth defects (occurring in about 1 in 1000 pregnancies, with marked ethnic and geographic variation in prevalence [12]). A few case reports can establish a strong association if a drug is taken by a relatively small number of women, causes a characteristic or obvious pattern of abnormalities (as is the case with most teratogens, for example thalidomide, warfarin and retinoic acid), or results in a rare malformation [13]. However, reports of common defects may reflect either background occurrence of these malformations in the general population or an increased risk for drug-induced birth defects. Moreover, without knowing the denominator (the total number of infants exposed to EFV in the first trimester of pregnancy), the relative risk of exposure is unknown.

Studies with prospectively followed pregnancies are subject to fewer biases than retrospective case reports. Enrolment in these studies occurs before the outcome of pregnancy is known and prior to tests that could provide knowledge of pregnancy outcome, such as antenatal ultrasound or alpha-fetoprotein measurement. These studies have been used to support a change in the United States Federal Drug Administration (FDA) pregnancy risk category, for example acyclovir changed to category B: "Positive animal data but adequate and well-controlled studies in humans failed to show a fetal risk".

In an prospective antiretroviral pregnancy registry based in the United States, birth defects were observed in 5 of 228 (2.2%; 95% CI: 0.7%-5.1%) live-born infants following first-trimester exposure to EFV and in 1 of 14 live births with second- or third-trimester exposure [14]. This prevalence of birth defects is comparable to the United States general population (3.1%; 95% CI: 3.1%-3.2%) [15]. The European Collaborative Study also collects data on pregnancy outcomes following ARV exposure during pregnancy. Thus far, 19 women in this study have become pregnant while receiving EFV-containing regimens, no congenital abnormalities were reported (0%; 95% CI: 0%-17.6%) [16]. In contrast to findings of the United States registry and European Collaborative Study, in a French cohort three of ten infants born to women who became pregnant while receiving EFV had birth anomalies [17]. None of the prospectively reported anomalies in the United States antiretroviral pregnancy registry or the French cohort were similar to those in the animal study or case reports.

A sufficient number of live births have been monitored in the United States antiretroviral pregnancy registry to detect a two-fold increase in overall risk for birth defects following first-trimester exposure to EFV; no such increase has been detected [14,18]. Several features of the registry limit the ability to draw definitive conclusions. Of eligible woman-infant pairs, only about 15% are enrolled [14]. It is unknown whether those not enrolled are at higher or lower risk of birth defects. Moreover, ascertainment of birth defects is not standardised, with varying use of diagnostic tests and level of expertise of reporting clinicians. Nevertheless, this evidence does provide some assurance that EFV is not a major human teratogen [19]. In sum, these findings indicate that any overall increase in risk for birth defects following exposure to EFV is likely to be low. However, larger studies are required to exclude an increased risk for specific congenital anomalies such as neural tube defects; current prospective studies have inadequate power to draw conclusions about the risk of neural tube defects [19].

## Efavirenz use in women of childbearing potential

In the FDA classification EFV is a category D drug: "Positive evidence of human fetal risk. Nevertheless, potential benefits may outweigh the potential risks" [20]. This disclaimer is understandable from a medicolegal standpoint, but provides no practical information for deciding whether potential benefits to a woman outweigh risks to a fetus or how to respond to inadvertent fetal exposures [21]. Furthermore, several critics argue that drugs are commonly assigned high-risk FDA categories based on limited information [13,21].

Two commonly used ARV treatment guidelines, developed by WHO and the United States Public Health Service Task Force, both recommend that EFV be avoided among women trying to conceive or not using contraception [22,23]. However, they differ for women using contraception. WHO guidelines indicate that EFV is a viable option for women using effective contraception [23], whereas guidelines from the United States recommend alternatives to EFV should be strongly considered because of known failure rates of contraception [22]. These guidelines target different settings with considerable variation in availability of ARV treatment options, which may account for differing recommendations.

Based on advantages of EFV and that existing data indicates any increase in overall risk for birth defects is likely to be small, withholding EFV-based treatment from women using contraception in settings with limited ARV options is likely to cause more harm than its provision. Further, safety advantages of EFV are particularly important in many high HIV burden settings with limited capacity for clinical and laboratory monitoring.

Withholding such treatment is contrary to principles guiding use of other drugs essential for a woman's health such as antiepileptic medication [24,25]. Much evidence indicates that carbamazepine and sodium valproate increase risk for neural tube defects [26,27], but in view of the need for effective control of seizures, recommendations are that in almost all cases, the optimum drug for controlling seizures should be used [24,25]. Principles guiding care for women with epilepsy share commonalities with those guiding ARV treatment decisions and could assist policy makers in selecting ARV regimens for women. This comparison applies particularly in settings with limited alternative drugs, while is less relevant to high-income countries with increased ARV options.

Analogous to antiepileptic medication, benefits of ARV treatment accrue both to the woman and to her fetus, and are likely to outweigh potential harm to the fetus. ARV treatment for women reduces mortality and morbidity, is the most effective method of preventing HIV transmission to the infant, and by securing the health of women, improves child survival [23,28]. On the basis of available evidence, several authors argue that decisions to initiate ARV treatment should be based primarily on a woman's need for such treatment [23,29,30].

Risk of unintended pregnancy is low with correct and consistent use of contraception [31]. However, evidence that EFV increases bioavailability of steroid hormones in hormonal contraceptives must be considered when selecting a contraceptive method [32]. Increased bioavailability may increase risk for estrogen- or progestin-related side effects. Alternative contraceptive methods with low typical-use failure rates need to be considered [31].

Women receiving EFV-containing regimens may later plan to become pregnant or have an unintended pregnancy [33]. For women who plan conception, substitution with NVP or a PI needs to be considered, although risks and benefits of substitution should be taken into account (Box 1). Drug substitution is best undertaken prior to pregnancy.

Some studies suggest teratogenic activity of drugs that increase risk for neural tube defects is mediated by interference with folic acid metabolism and that folic acid supplementation protects against teratogenic effects of these drugs [34,35]. Although potential mechanisms of EFV-induced fetal effects are unknown, folic acid supplementation is important for women receiving EFV as it is for other women of childbearing age.

## Use of efavirenz during pregnancy

Pregnancy recognition often occurs after the critical period of organogenesis (3–8 weeks post-conception). Development and closure of the neural tube are normally complete by 28 days post-conception, approximately the same gestation when the first symptoms of pregnancy occur. Changes to EFV-based regimens after four weeks post-conception will not reduce the risk of neural tube defects and after eight weeks will have minimal effect on risk for other structural malformations. There are theoretical risks that exposure to EFV or other ARV drugs in the second and third trimester of pregnancy could affect neurodevelopment of infants. However, in the absence of evidence from neurobehavioral development studies, effects of exposure to any ARV drug after the period of organogenesis remain speculative.

Women who realise they are pregnant early in gestation can consider substituting EFV with NVP or a PI, or temporarily suspending treatment. Substituting EFV with another ARV drug is commonly considered, but is not without risk [23] (Box 1).

**Box 1**. Factors to consider when substituting efavirenz in pregnant women:

• Following substitution, women have to get used to a new ARV regimen, with different side-effects, pill counts and dosing times;

• Treatment-related increases in CD4 cell count may have occurred. Substituting EFV with NVP in women with treatment-related CD4 cell restoration to levels above 250 cells/mm^3 ^could, in theory, place them at increased risk for NVP-associated hepatotoxicity;

• In many settings alternative ARV drugs are limited by availability, cost or co-existing conditions; and

• Substituting EFV with other drugs may limit the effectiveness of future regimens. Pharmacokinetic evidence suggests that when substituting EFV with NVP, women should commence on 200 mg twice a day, as dose escalation of NVP is associated with sub-therapeutic NVP levels in these individuals [36].

Temporarily suspending ARV treatment also has several risks. Suspending ARV treatment at recognition of pregnancy has been associated with significant viral rebound and CD4 cell count decline [37], potentially increasing risk for HIV transmission to the fetus and compromising a woman's health. In addition, EFV has a longer half-life than nucleoside analogue reverse transcriptase inhibitor (NRTI) drugs resulting in functional monotherapy, which increases risk for viral resistance [38,39]. Many experts recommend continuing the NRTI backbone for a period of time after NNRTI discontinuation [5,22]. Although evidence is accumulating [40,41], the optimal interval between stopping NNRTI and other ARV drugs is unknown.

Women who become pregnant while receiving EFV require counselling and full information on potential risks to the fetus. High-quality counselling entails non-directive individualised discussion of options and support for a woman to make an autonomous decision about use of ARV drugs or termination of pregnancy, to the extent allowed by law [42]. It is important to note that an exaggerated perception of fetal risk can result in a woman terminating an otherwise wanted pregnancy [13]. Such decisions are complex and underpinned by biomedical as well as socio-cultural considerations.

Women who become pregnant while receiving EFV may benefit from screening for CNS abnormalities with a fetal anomaly ultrasound or maternal serum alpha-fetoprotein test [43]. These non-invasive tests are preferable as amniocentesis has been associated with increased risk for HIV infection in infants [44].

## Efavirenz for preventing HIV infection in infants

Advocacy is mounting for prevention of mother-to-child transmission (MTCT) programmes in resource-constrained settings to introduce more effective ARV prophylaxis than single-dose (maternal and infant) NVP [45,46]. Evidence is accumulating of the feasibility of providing triple-ARV regimens for prophylaxis in resource-constrained settings [47,48]. In Brazil, Europe and the United States, for a woman without indications for ARV treatment, triple-ARV prophylaxis is provided during pregnancy and discontinued after childbirth [22,49,50], and the risk of transmitting HIV to her infant is less than 2% [51,52]. Triple-ARV prophylaxis is used for almost all pregnant women with HIV in these settings. For example, United States MTCT-prevention guidelines state: "Standard combination antiretroviral regimens for treatment of HIV-1 infection should be discussed and offered to all pregnant women with HIV-1 infection regardless of viral load; they are recommended for all pregnant women with HIV-1 RNA levels greater than 1000 copies/mL" [22].

MTCT-prophylaxis regimens are initiated after the first trimester of pregnancy, hence past the period of organogenesis. An EFV-containing triple ARV regimen could be a useful alternative for MTCT-prevention programmes that adopt similar strategies to Brazil, the United States and Europe. Based on WHO guidelines, EFV-based triple regimens are a viable option for MTCT prophylaxis [23]. Alternative triple regimens for pregnant women without indications for ARV treatment pose several difficulties: women with a high CD4 cell count have an increased risk for hepatotoxicity with NVP; and protease inhibitor containing regimens have a higher pill burden, more complex side-effect profile and higher cost.

## Conclusion

Existing ARV treatment guidelines do not adequately address the complex clinical scenarios that women and clinicians increasingly face. This has compounded difficulties in making the inevitable trade-offs between reproductive and treatment choices. Based on existing evidence we have outlined general considerations in these scenarios. In particular, as described in current WHO guidelines for resource-constrained settings, the benefits of EFV are likely to outweigh risks for women using contraception. However, we argue that in women who become pregnant while receiving EFV, a decision to temporarily suspend treatment or to substitute EFV after the period of organogenesis is unwarranted, especially in settings with limited alternative drugs. Moreover, given limitations of existing data, additional evidence is needed to assist individual patients to balance risks and benefits.

Several research centres in Africa have the capacity to recruit an adequate number of exposed woman-infant pairs and to ensure high rates of cohort retention and accurate ascertainment of pregnancy outcomes. With the rapid increase in women receiving ARV treatment in Africa and as EFV is the preferred NNRTI in many settings, these centres are ideally situated to establish an adequately powered ARV registry. Additional scientifically valid data and estimates of relative risk would provide more detailed information of potential risks, or conversely offer further reassurance that EFV and other ARV drugs are not major human teratogens. Without this evidence, creating policy consensus is difficult and women will continue to face reproductive decisions with limited information.

## Competing interests

The author(s) declare that they have no competing interests.

## Authors' contributions

MC conceived of the review and with MU drafted the manuscript. WV wrote sections of the review. TW, AK, GG, SL and DL made substantial contributions to content of the paper.
